# A real-world retrospective study to assess efficacy and safety of alectinib as adjuvant therapy in IB-IIIB NSCLC patients harboring *ALK* rearrangement

**DOI:** 10.3389/fonc.2024.1422035

**Published:** 2024-10-21

**Authors:** Zeng-Hao Chang, Teng-Fei Zhu, Wei Ou, Hao Jiang, Si-Yu Wang

**Affiliations:** Department of Thoracic Surgery, Sun Yat-sen University Cancer Center, State Key Laboratory of Oncology in South China, Collaborative Innovation Center for Cancer Medicine, Guangzhou, China

**Keywords:** adjuvant therapy, anaplastic lymphoma kinase-tyrosine kinase inhibitor, alectinib, NSCLC, dfs

## Abstract

**Background:**

Alectinib has demonstrated promising disease-free survival (DFS) benefit for early-stage non-small cell lung cancer (NSCLC) patients with ALK rearrangement positive in phase 3 ALINA trial. However, real-world evidence for the efficacy and safety of alectinib in early-stage ALK-positive NSCLC is limited.

**Materials and methods:**

We retrospectively reviewed 68 patients with stage IB-IIIB ALK-positive NSCLC who underwent complete pulmonary resections from April 2010 to July 2023 at a single institution. 38 (55.9%) enrolled patients had N2 lymph node metastasis, and 17 (24.9%) patients had multi-station N2 metastasis. Patients were stratified into two groups according to the adjuvant treatment regimen, with 19 patients in the alectinib group and 49 patients in the chemotherapy group. There were no significant differences in clinicopathological characteristics between the two groups. After curative resection surgery, patients in alectinib group received oral alectinib at a dose of 600 mg twice daily and patients in chemotherapy group received platinum-based doublet chemotherapy regimen every 3 weeks for 4 cycles. The primary endpoint was 3-year DFS. The Kaplan-Meier method was used to estimate DFS and overall survival (OS). Safety analyses were conducted by comparing the incidence of adverse events between the two groups.

**Results:**

At the last follow-up date (January 22th, 2024), A total of 1 (5.3%) and 28 (57.1%) DFS events were observed in alectinib group and chemotherapy group respectively. The 3-year DFS showed significant improvement in the alectinib group compared with chemotherapy group (91.7% vs 60.7%, P=0.051). In the IIIAN2 subgroup, the 3-year DFS rate in the alectinib group reached a satisfactory 87.5%. In both groups, the majority of AEs were graded as level 1 or 2, No grade 3-4 AEs were observed in alectinib group.

**Conclusion:**

Alectinib, as adjuvant therapy, demonstrated favorable efficacy and manageable safety in patients with completely resected ALK-positive stage I B-IIIB non-small cell lung cancer. A limitation of this study is the small sample size, and a larger-scale real-world sample study is needed to further evaluate the efficacy and safety of alectinib as adjuvant therapy.

## 1 Introduction

With an estimated 12.8 million deaths per year, lung cancer remains one of the most frequently diagnosed cancers and the leading cause of cancer-related mortality worldwide (1). Around 85% of lung cancers are non-small cell lung cancer (NSCLC) (2). Surgical resection remains the most effective treatment option in patients with stage I-IIIA NSCLC (3), unfortunately, approximately 60-70% of patients subsequently develop relapse or metastasis after radical resection, which are the main reasons for the poor prognosis of NSCLC. Moreover, With the increase in the number and station of metastatic lymph nodes, the 5-year overall survival rate (OS) decreases significantly. Therefore, postoperative systemic therapy is of great significance to improve the survival rate of NSCLC patients. Adjuvant (or postoperative) chemotherapy has demonstrated the potential to improve survival in patients with early-stage NSCLC following complete resection, nevertheless, the observed survival outcomes remain suboptimal (4–6).

The introduction of molecular targeted therapies has transformed the treatment and prognosis of patients with NSCLC by integrating tumor genetic mutations into therapeutic decision making (7, 8). Previous studies have confirmed the efficacy of adjuvant TKIs for respectable NSCLC with targetable driver mutations (9–11). The ADJUVANT, EVAN, and ADAURA trails have similarly confirmed that adjuvant EGFR-TKI therapies could provide greater benefits for EGFR-positive NSCLC patients in terms of DFS compared to adjuvant chemotherapy. Nonetheless, most of the data available on adjuvant TKI therapies are regarding EGFR-positive NSCLC patients (12).

The rearrangements of anaplastic lymphoma kinase (ALK) gene occur in approximately 3-5% of NSCLC patients and ALK-positive NSCLC patients are highly sensitive to ALK receptor tyrosine kinase inhibitors (TKIs) (2, 13, 14). ALK-TKIs as first-line treatment for advanced ALK-positive NSCLC patients have been abundantly evaluated and the superior efficacies were established (15–17). Alectinib, a second-generation highly selective ALK-TKI, has shown improved efficacy and tolerability compared with the first-generation ALK-TKI for the treatment of advanced ALK-positive NSCLC in both ALEX and ALISIA trials (18, 19). The ALINA study, a groundbreaking prospective clinical trial, represents a significant advancement in the field of adjuvant therapy for ALK-positive NSCLC. Compared to chemotherapy, alectinib stands out as the pioneering ALK inhibitor that notably enhances disease-free survival (DFS) in postoperative patients diagnosed with IB-IIIA ALK-positive NSCLC (20). As the findings from controlled clinical trials may not always fully reflect the diverse patient populations encountered in real-world clinical practice, this retrospective analysis aims to complement and extend the evidence from the ALINA study by investigating the real-world effectiveness and safety of alectinib as adjuvant therapy for NSCLC patients.

## 2 Materials and methods

### 2.1 Patients

This retrospective study included patients with ALK-positive NSCLC who underwent different types of curative pulmonary resections between April 2010 and July 2023 at Sun Yat-sen University Cancer Center in China. Before surgery, each patient was routinely evaluated through positron emission tomography-computed tomography (PET-CT) or intravenous contrast-enhanced CT scans of the chest and abdomen, brain magnetic resonance imaging (MRI), bronchoscopy, and bone scan. Patients received objectives sublobectomy, lobectomy or Pneumonectomy resection with lymphadenectomy, depending on whether the lung lesions were completely removed during surgery. Patients were considered eligible for inclusion in the study if they: were 18 years of age or older, had an Eastern Cooperative Oncology Group (ECOG) performance status of 0 to 1, had completely resected (R0) stage IB to IIIB disease (according to the 8th edition of the AJCC TNM staging system for lung cancer (21)), had a cytologically or histologically proven *ALK* gene rearrangement through the method of fluorescence *in situ* hybridization (FISH), received alectinib or platinum-based chemotherapy as adjuvant therapy, had adequate hematological function, liver function and renal function. Patients were excluded if they had: previous exposure to other targeted agents (eg, crizotinib, ensartinib or brigatinib); radiotherapy before or after surgery; pulmonary resections for palliative purposes; occurrence of a second primary malignancy either prior to or during the study period.

### 2.2 Treatment methods

A total of 68 ALK-positive NSCLC patients who underwent curative surgery were enrolled in this retrospective study. The patients were divided into two groups, consisting of 19 cases in the alectinib group and 49 cases in the chemotherapy group. In the chemotherapy group, 49 patients diagnosed with adenocarcinoma underwent a 4-cycle treatment consisting of pemetrexed plus cisplatin or carboplatin (AP regimen), while patients with squamous cell carcinoma received a 4-cycle treatment of pemetrexed plus cisplatin (PP regimen). Patients who experienced recurrence in the chemotherapy group subsequently underwent additional therapeutic interventions, including targeted therapy, radiation therapy, or enrollment in ongoing clinical trials. In the alectinib group, patients voluntarily received oral alectinib at a dose of 600 mg twice daily after resection. The alectinib therapy continued until disease progression (either radiographic or clinical confirmation) or severe toxicity was observed. The study was conducted in accordance with the Declaration of Helsinki and was approved by the Ethics Committee of Sun Yat-Sen University Cancer Center. As adjuvant treatment with alectinib has not yet been approved in China, all patients receiving alectinib treatment have signed informed consent forms for off-label use.

### 2.3 Follow up and assessment

Patients were regularly followed up every 3-6 months after surgery. Disease recurrence was evaluated based on tumor assessments at follow-up visits, including enhanced CT every 3 or 6 months and brain MRI every 1 year (or as indicated based on symptoms.) Tumor responses were assessed by Response Evaluation Criteria in Solid Tumors (RECIST version 1.1). To evaluate cancer recurrence, we defined locoregional recurrence as recurrence at the lung, mediastinal, or supraclavicular lymph nodes. Systemic recurrence was identified by recurrence at sites such as the pleura, pericardium, and extrathoracic sites like the brain, liver, or bone (22). The primary endpoint was 3-year DFS, defined as the proportion of patients who remained free of disease at the 3-years. The secondary endpoints included DFS (time from the date of surgical resection until the first documented evidence of relapse or death from any cause), overall survival (OS, time from the date of surgical resection to death from any cause), 5-year OS (the proportion of patients still alive 5 years after surgery), and safety. The AEs were evaluated according to National Cancer Institute Common Terminology Criteria for Adverse Events (NCI CTCAE), version 4.0. All the follow-up data were collected via medical records of patients at hospital and phone calls to the patients or their relatives.

### 2.4 Statistical analyses

The characteristics of patients were summarized with descriptive statistics. Continuous variables (such as age) were presented as mean (± standard deviation [SD]) for normally distributed data, and comparisons were made using t-tests. Categorical variables (such as gender, stage, etc.) were presented as number (percentage), and group comparisons were performed using chi-square tests or Fisher’s exact tests. The point estimates of 3-year DFS and 5-year OS in the alectinib and chemotherapy groups were calculated using the Kaplan-Meier method. The estimates of the median DFS and OS were calculated by the Kaplan–Meier method and Kaplan–Meier curves were depicted. Survival outcomes were compared with the log-rank test. P values < 0.05 were considered statistically significant. Cox proportional hazards models were used to estimate HRs with their 95% confidence intervals (CIs). R (4.3.0, R Foundation, Vienna, Austria) was used to perform the statistical analyses.

## 3 Results

### 3.1 Patient characteristics

Between April 2010 and July 2023, a total of 68 patients who received alectinib or chemotherapy as adjuvant therapy were enrolled in this retrospective study. The clinicopathological characteristics of patients are shown in **Table 1**. The mean age of the population was 53.0 (± 10.7) years. 32 (47.1%) patients were females and 36 (52.9%) patients were male. 46 (67.6%) patients were non-smokers. Regarding the histological type, 64 (94.1%) patients were adenocarcinoma, 1 (1.5%) patient was Squamous cell carcinoma, and 3 (4.4%) patients were Adenosquamous carcinoma. Among the enrolled patients, 10 (14.7%) patients were in stage IB, 3 (4.4%) patients were in stage IIA,16 (23.5%) patients were in stage IIB, 36 (52.9%) were in stage IIIA and 3 (4.4%) were in stage IIIB NSCLC. 64 (94.1%) patients underwent lobectomy, 3 (4.4%) patients underwent sublobectomy, and 1 (1.5%) patient underwent Pneumonectomy. Concerning the involvement of mediastinal lymph nodes, 38 (55.9%) patients exhibited N2 lymph node metastasis, and 27 (39.7%) patients had involvement of three or more mediastinal lymph nodes.

**Table 1:** Clinicopathological Characteristics of patients in the chemotherapy and alectinib group.

	Chemotherapy group (n=49)	Alectinib group (n=19)	Total (n=68)	P value
Age, yrs				0.375
Mean (SD)	52.3 (10.9)	54.8 (10.5)	53.0 (10.7)	
Gender				0.098
Female	20 (40.8%)	12 (63.2%)	32 (47.1%)	
Male	29 (59.2%)	7 (36.8%)	36 (52.9%)	
Smoking Status				0.215
Never smoker	31 (63.3%)	15 (78.9%)	46 (67.6%)	
Smoker	18 (36.7%)	4 (21.1%)	22 (32.4%)	
ECOG				0.130
0	48 (98.0%)	17 (89.5%)	65 (95.6%)	0.384
1	1 (2.0%)	2 (10.5%)	3 (4.4%)	
Tumor Location				0.066
Left lower lobe	8 (16.3%)	9 (47.4%)	17 (25.0%)	
Left upper lobe	9 (18.4%)	2 (10.5%)	11 (16.2%)	
Right lower lobe	16 (32.7%)	6 (31.6%)	22 (32.4%)	
Right middle lobe	3 (6.1%)	1 (5.3%)	4 (5.9%)	
Right upper lobe	13 (26.5%)	1 (5.3%)	14 (20.6%)	
Surgery type				0.015
Sublobectomy	0 (0.0%)	3 (15.8%)	3 (4.4%)	
Lobectomy	48 (98.0%)	16 (84.2%)	64 (94.1%)	
Pneumonectomy	1 (2.0%)	0 (0.0%)	1 (1.5%)	
T stage				0.197
T1	20 (40.8%)	4 (21.1%)	24 (35.3%)	
T2	26 (53.1%)	13 (68.4%)	39 (57.4%)	
T3	3 (6.1%)	1 (5.3%)	4 (5.9%)	
T4	0 (0.0%)	1 (5.3%)	1 (1.5%)	
Lymph node status				0.382
N0	11 (22.4%)	2 (10.5%)	13 (19.1%)	
N1	13 (26.5%)	4 (21.1%)	17 (25.0%)	
N2	25 (51.0%)	13 (68.4%)	38 (55.9%)	
Stations of metastatic N2 lymph nodes				0.734
0	21 (42.9%)	7 (36.8%)	28 (41.2%)	
1	16 (32.7%)	7 (36.8%)	23 (33.8%)	
2	8 (16.3%)	4 (21.1%)	12 (17.6%)	
3	3 (6.1%)	0 (0.0%)	3 (4.4%)	
4	1 (2.0%)	1 (5.3%)	2 (2.9%)	
No. of metastatic lymph nodes				0.421
≥3	18 (36.7%)	9 (47.4%)	27 (39.7%)	
0-2	31 (63.3%)	10 (52.6%)	41 (60.3%)	
Histologic type				0.439
Adenocarcinoma	45 (91.8%)	19 (100.0%)	64 (94.1%)	
Adenosquamous carcinoma	3 (6.1%)	0 (0.0%)	3 (4.4%)	
Squamous cell carcinoma	1 (2.0%)	0 (0.0%)	1 (1.5%)	
Differentiation				0.061
Low	12 (24.5%)	11 (57.9%)	23 (33.8%)	
Moderate	9 (18.4%)	3 (15.8%)	12 (17.6%)	
Moderate/High	1 (2.0%)	0 (0.0%)	1 (1.5%)	
Moderate/Low	27 (55.1%)	5 (26.3%)	32 (47.1%)	
stage				0.397
IB	9 (18.4%)	1 (5.3%)	10 (14.7%)	
IIA	2 (4.1%)	1 (5.3%)	3 (4.4%)	
IIB	12 (24.5%)	4 (21.1%)	16 (23.5%)	
IIIA	25 (51.0%)	11 (57.9%)	36 (52.9%)	
IIIB	1 (2.0%)	2 (10.5%)	3 (4.4%)	
Comparison of first site of metastasis				<0.001
No	21 (42.9%)	18 (94.7%)	39 (57.4%)	
Locoregional	10 (20.4%)	1 (5.3%)	11 (16.2%)	
Systemic	18 (36.7%)	0 (0.0%)	18 (26.5%)	
Death				0.017
No	37 (75.5%)	18 (94.7%)	55 (80.9%)	
Yes	12 (24.5%)	1 (5.3%)	13 (19.1%)	

(n=68) ECOG, Eastern Cooperative Oncology Group.

### 3.2 Efficacy

**Figure 1:**
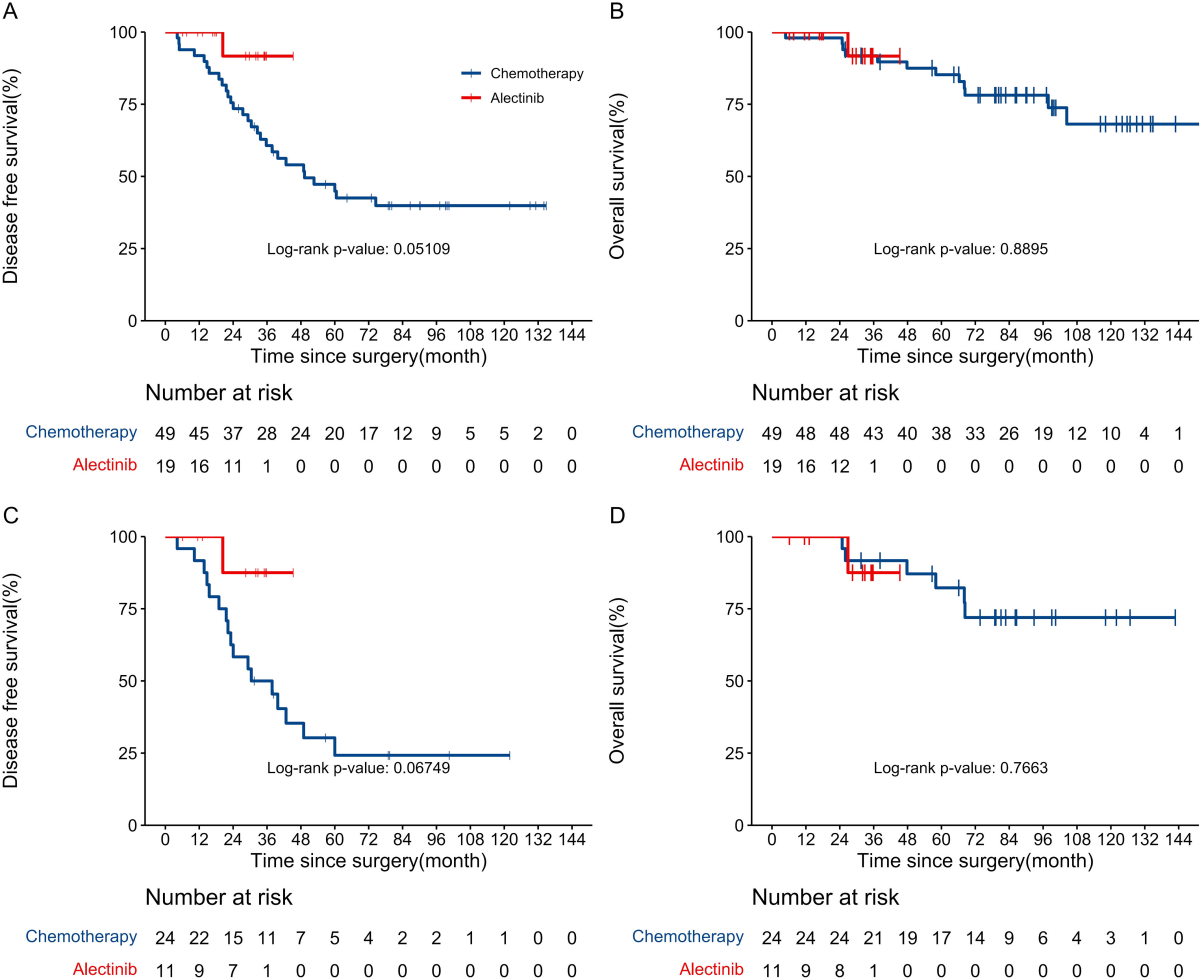
**(A)** Kaplan–Meier curves for disease-free survival between chemotherapy and alectinib group. **(B)** Kaplan–Meier curves for overall survival between chemotherapy and alectinib group. **(C)** Kaplan–Meier curves for disease-free survival of IIIAN2 patients between chemotherapy and alectinib group. **(D)** Kaplan–Meier curves for overall survival of IIIAN2 patients between chemotherapy and alectinib group.

At the last follow-up date (January 22nd, 2024), a total of 29 DFS events and 12 OS events were observed in two groups. The median follow-up for DFS was 35.1 (IQR 12.5–57.7) months, while the median follow-up for OS was 68.2 (IQR 35.5–101.0) months. In the alectinib group, the DFS and OS events were observed in only 1 (5.3%) of the 19 patients. This patient received pulmonary sublobectomy and Mediastinal lymph node dissection in February 2019, with a pathologic diagnosis of stage IIIA (pT1bN2M0) poorly differentiated invasive lung adenocarcinoma. Then, the patient received alectinib as adjuvant therapy and experienced a recurrence of the primary lesion and mediastinal lymph node metastasis in the 20th month of alectinib treatment, the patient subsequently joined a phase I clinical trial with the novel FAK/ALK inhibitor APG-2449 and took APG-2449 at a dose of 450 mg QD. Six months after starting APG-2449, the patient developed a pericardial effusion and ultimately died seven months later. No patient in alectinib group experienced central nervous system progression or relapse during alectinib treatment. In the chemotherapy group, recurrence occurred in 28 (57.1%) patients, while mortality was observed in 12 (24.5%) patients. The Kaplan-Meier curves illustrating the probability of DFS and OS for patients are presented in **Figures 1A, B**. The 3-year DFS was significantly higher in the alectinib group (91.7%, 95% CI, 77.3%–100.0%) than in the chemotherapy group (60.7%, 95% CI, 48.3%–76.2%). And it is shown that the DFS were significantly improved in the alectinib group compared with chemotherapy group (HR 0.17; 95% CI, 0.02–1.28; P = 0.051). The HR equaled an 83% reduction in the risk of disease recurrence or death. Although the P-value of 0.051 is marginally above the conventional significance threshold of 0.05, it may still suggest a trend toward alectinib reducing the risk of recurrence or death. The relatively wide confidence interval (0.02 to 1.28) is likely a result of the small sample size in the alectinib group, which increases the variability of the effect estimate. Despite this limitation, the HR indicates a substantial reduction in the risk associated with alectinib treatment, which could have clinical relevance. The 5-year OS rate was not reached in alectinib group and 85.2% (95% CI, 75.7%–76.0%) in chemotherapy group. The OS did not show a statistically significant difference between the alectinib and chemotherapy groups. The HR for alectinib was 0.8566 (95% CI, 0.10–7.63; P = 0.89), indicating a 14.3% reduction in the risk of death. However, the P-value of 0.89 exceeds the conventional significance threshold of 0.05, suggesting no significant differences in survival outcomes. The wide confidence interval (0.10 to 7.63) indicates considerable uncertainty, likely due to the limited sample size and few observed events. Additionally, 17 (63.0%) of the 27 patients in the chemotherapy group received targeted therapy with ALK inhibitors after recurrence, which may have affected the overall survival results. We further conducted an analysis of DFS and OS among patients in the subgroup with stage IIIA and N2 involvement. The Kaplan-Meier curves illustrating the probability of DFS and OS for IIIAN2 patients are presented in **Figures 1C, D**. The 3-year DFS rates were 87.5% (95% CI, 67.3%–100.0%) in the alectinib group and 50.0% (95% CI,33.5%–74.6%) in the chemotherapy group. The Kaplan-Meier curves for DFS indicate a favorable trend in the alectinib group compared to the chemotherapy group. The log-rank test for DFS analysis yielded a p-value of 0.068, which is marginally above the traditional significance threshold of 0.05. The HR for alectinib treatment was 0.18 (95% CI, 0.02–1.41), suggesting an approximate 82% reduction in the risk of disease recurrence compared to chemotherapy. These findings imply that alectinib may be associated with improved DFS, highlighting its potential clinical relevance. For OS, the analysis yielded a p-value of 0.768, indicating no statistically significant difference between the alectinib and chemotherapy groups. The HR for alectinib treatment was 1.43 (95% CI, 0.13–15.85), suggesting a potential increase in risk, but the wide confidence interval limits the interpretability of this finding. The 5-year OS rate was not reached in the alectinib group, while the chemotherapy group had an OS rate of 82.2% (95% CI, 67.8%–99.7%). Given these results, further follow-up studies are needed to evaluate the long-term treatment responses in the 19 patients of the alectinib group.

### 3.3 Safety

In comparison to the chemotherapy group, patients in alectinib group exhibited a lower occurrence of adverse events across any severity levels. (**Table 2**) In both groups, the majority of adverse events were graded as level 1 or 2. The most common grade 1-2 adverse events for which the incidence was at least 5 percentage points higher in the alectinib group than in the chemotherapy group were constipation (occurring in 47% of the patients), Weight increased (in 21%), Rash (in 16%), and Musculoskeletal pain (in 11%). In the chemotherapy group, the most common grade 1-2 adverse reactions are Nausea and vomiting (63%), followed by Alopecia (47%) and ALT/AST increased (39%). Grade 3 or 4 AEs occurred in 2 patients (4%) in the chemotherapy group, including Neutropenia in 2 patient (4%), Lymphopenia in 1 patient (2%), Nausea and vomiting in 1 patient (2%). There was no Grade 3 or 4 adverse event occurred in patients from the alectinib group. The alectinib treatment did not result in any unanticipated AEs and there were no deaths attributed to the treatment.

**Table 2:** Adverse events in the chemotherapy and alectinib groups.

	Chemotherapy group (n=49)		Alectinib group (n=19)	
	Grade 1-2	Grade 3-4	Grade 1-2	Grade 3-4
Total adverse events	41 (84%)	2 (4%)	9 (47%)	0
ALT/AST increased	19 (39%)	0	0	0
Alopecia	23 (47%)	1 (2%)	2 (11%)	0
Anemia	19 (39%)	0	0	0
Constipation	18 (37%)	0	9 (47%)	0
Diarrhea	7 (14%)	0	3 (16%)	0
Dysgeusia	9 (18%)	0	0	0
ECG Q-T prolonged	1 (2%)	0	0	0
Interstitial pneumonia	1 (2%)	0	0	0
Lymphopenia	15 (31%)	1 (2%)	0	0
Musculoskeletal pain	1 (2%)	0	2 (11%)	0
Nausea and vomiting	31 (63%)	1 (2%)	0	0
Neutropenia	15 (31%)	2 (4%)	0	0
Peripheral edema	4 (8%)	0	0	0
Photosensitivity reaction	0	0	0	0
Pulmonary embolism	0	0	0	0
Rash	3 (6%)	0	3 (16%)	0
SCr/BUN increased	2 (4%)	0	0	0
Trombocytopenia	7 (14%)	0	0	0
Visual impairment	2 (4%)	0	0	0
Weight increased	0	0	4 (21%)	0

Data are n (%). ALT, alanine aminotransferase; AST, aspartate aminotransferase; ECG, electrocardiogram; SCr, serum creatinine; BUN, blood urea nitrogen.

## 4 Discussion

Effective adjuvant targeting therapies have been extensively documented in postoperative NSCLC patients, including Osimertinib in the management of IB-IIIA EGFR-positive NSCLC patients (11). The rearrangement of the ALK gene occurs in approximately 3-5% of NSCLC patients and represent a distinct molecular subset of NSCLC (2, 23). To date, there is still no consensus on the postoperative treatment for ALK-positive NSCLC patients. In addition to the ALINA study, we are the first to report data on postoperative adjuvant treatment with alectinib in Chinese patients with ALK-positive NSCLC, demonstrating consistent efficacy and superior safety. In this retrospective study, we compared the effectiveness and safety of adjuvant alectinib and adjuvant chemotherapy in 68 ALK-TKI-naive patients with completely resected ALK-positive, stage IB to IIIB NSCLC. Our findings demonstrate that patients in alectinib group achieved a favorable 3-year DFS rate of 91.7%, significantly higher than the chemotherapy group, which had a 3-DFS of 60.7%. Furthermore, alectinib exhibited excellent tolerability in 19 patients as only mild adverse events were observed and no occurrences of severe adverse events or treatment interruption due to adverse effects. In summary, our study suggested that alectinib, as adjuvant therapy, holds promise for both clinical efficacy and safety in enhancing the prognosis of postoperative ALK-positive NSCLC patients.

Previous study indicated that ALK-positive lung adenocarcinoma patients exhibit distinct clinical characteristics. ALK-positive patients are typically younger and have a history of never or light smoking compared to those with ALK-negative lung adenocarcinoma (24, 25). Here, we showed that the mean age of 14 patients was 53.0 (± 10.7) years and 46 (67.6%) patients were never smokers. These results were basically consistent with data reported in a previous study by Shaw AT et al. (26). At present, the prognostic significance of ALK gene in postoperative non-small cell lung cancer patients remains controversial. A previous retrospective study had shown that ALK-rearrangement was associated with more frequent recurrence in patients with surgically resected early-stage NSCLC (27). Similar results were observed in local advanced patients in another study, in which EML4-ALK-positive patients of stage IIIA had poorer DFS than EML4-ALK-negative patients (median DFS 6 versus 16 months, P=0.0057) (28). In addition, ALK-positive adenocarcinomas tend to present a more rapid metastases to lymph nodes or distant sites compared with EGFR mutation or with wild type, indicating that the tumor is more aggressive (29, 30). Thus, postoperative therapy is indispensable for those subgroup of NSCLC patients. Till now, platinum-based chemotherapy remains the standard of care for postoperative treatment of IB-IIIB ALK-positive NSCLC patients (31). However, this treatment only reduced the risk of relapse or death by 16% and the risk of death was reduced by only 5% over 5 years (32, 33). The ALINA study is a randomized, actively controlled, multicenter, open-label phase III clinical trial aimed at investigating the efficacy and safety of alectinib as adjuvant therapy in postoperative ALK-positive NSCLC. Results from a 27.8-month follow-up revealed that the use of alectinib as adjuvant therapy in completely resected stage IB to IIIA ALK-positive NSCLC patients can reduce the risk of disease recurrence or death by 76% compared to the platinum-based chemotherapy group, (hazard ratio [HR] = 0.24, 95% CI: 0.13-0.43, p <0.0001). In the intention-to-treat (ITT) population (stage IB-IIIA), the 3-year disease-free survival (DFS) rate in the alectinib group reached 88.3%, closely aligning with the results observed in our real-world observations (20). Although the OS data for patients undergoing adjuvant alectinib in the ALINA study are still immature, a combined analysis with existing data, following extended follow-up, suggests a considerable improvement in total survival for early-stage ALK-positive NSCLC patients treated with alectinib.

N2-positive stage IIIA patients represent a heterogeneous group of locally advanced non-small cell lung cancer (NSCLC) (34). Till now, there is a lack of consensus on the appropriate therapeutic strategies for patients with IIIA-N2 NSCLC (35). Compared with early-stage patients, the recurrence rate of stage IIIA-N2 patients was significantly higher. Despite radical resection, reported 5-years survival rate of these patients was only 15–20% (36). The unsatisfactory survival rate of surgery has led to ongoing efforts to add non-surgery treatment strategies (37). Chemotherapy delivered as postoperative therapy may eradicate micro metastasis and reduce the onset of recurrence of NSCLC (38). A number of randomized controlled trials have been conducted in patients with stage IIIA-N2 NSCLC. Ou et al. and Daniel J. Boffa et al. have shown that postoperative cisplatin-based chemotherapy improved overall survival (OS) compared to surgery alone (39, 40). However, a pooled analysis revealed that adjuvant chemotherapy provides an unsatisfactory 17% reduction in the risk of death for stage III patients (33). The extent of mediastinal lymph node involvement is a significant prognostic factor for NSCLC (41). A retrospective study conducted by Andre et al. analyzed data on 702 patients with N2-NSCLC patients and concluded that the 5-year survival rates of patients were correlated with the degree of mediastinum involvement. The survival rate of patients with multi-station lymph nodes involvement was lower than that of patients with single-station lymph node involvement. (11%vs34%) (42). The number of regional lymph node is another leading prognostic factor of NSCLC patients (34). Patients with increased number of lymph nodes involved (specifically ≥3 LNs) experienced bad prognosis (43–45). In our study, patients with stage IIIA/N2 experienced a 3-year DFS of 87.5%. It was worth noting that 26.4% of enrolled patients had multi-station lymph nodes involvement and 47.4% of patients and at least three Metastatic lymph nodes. Our result indicated that adjuvant alectinib may be a better option for resected IIIA/N2 ALK positive NSCLC patients compared with chemotherapy. Despite the short follow-up period and limited number of patients in our study, the efficacy results support a further and large-sample investigation.

Brain metastasis is one of the common metastasis sites in NSCLC and usually indicates a poor prognosis (46). Up to 50-60% of patients with ALK-positive NSCLC are likely to develop brain metastases (BMs) throughout the course of disease (47). With the ability of crossing the blood–brain barrier (48–50), alectinib has demonstrated an indisputable superiority compared to crizotinib in intracerebral disease control (51). In the ALEX trial, The time to CNS progression was significantly longer with alectinib than with crizotinib (cause-specific hazard ratio, 0.16, 95% CI, 0.10 to 0.28; rate of events of CNS progression, 12% with alectinib and 45% with crizotinib) (18). Similar to excellent results achieved in advanced patients, none of patients enrolled in our study had CNS metastasis during the treatment of alectinib, which indicated that adjuvant alectinib may reduce the risk of CNS recurrence among patients with resected ALK-positive NSCLC.

The type and grade of AEs observed in this study were in accordance with the known safety profile of alectinib in advanced NSCLC. Alectinib was well-tolerated in study patients and no discontinuation or dose interruption due to adverse events were emerged. The incidence of overall drug-related AEs was 47.0%. However, the incidence of Grade 3-5 AEs in our study is far less than which observed in both ALEX and ALESIA trials (0% vs 28% and 29%) (18, 19). The possible reason could be that patients in the ALEX and ALESIA trials had advanced stage NSCLC hence having poorer physical conditions and higher tumor burdens than resected patients.

There were several limitations to this study. Selection bias was inevitable due to the inherent limitations of single-center, nonrandomized and retrospective design. Small sample size and short follow-up period led to insufficient data and immature results. Another important consideration is the subsequent use of ALK inhibitors in the chemotherapy group after recurrence, which could have confounded the OS results, potentially diluting the observed effect of adjuvant alectinib. The lack of randomization further limited the ability to control for confounding factors, such as differences in baseline characteristics between the treatment groups. Therefore, more prospective studies with longer follow-up time are needed to further prove the efficacy and safety of adjuvant alectinib in patients with ALK-positive NSCLC patients.

## 5 Conclusions

In conclusion, our retrospective study indicates that adjuvant alectinib is a promising therapeutic strategy for improving DFS in patients with ALK-positive lung cancer and has a favorable safety profile. Although the 5-year OS data have not yet been reached, these findings suggest the potential of alectinib to enhance patient prognosis. However, the limited availability of clinical data highlights the necessity for further studies to establish a standardized treatment regimen for postoperative ALK-positive NSCLC.

## Data Availability

The raw data supporting the conclusions of this article will be made available by the authors, without undue reservation.

## References

[1] Sung H Ferlay J Siegel RL Laversanne M Soerjomataram I Jemal A. Global cancer statistics 2020: GLOBOCAN estimates of incidence and mortality worldwide for 36 cancers in 185 countries. CA Cancer J Clin. (2021) 71:209–49. doi: 10.3322/caac.21660 33538338

[2] Thai AA Solomon BJ Sequist LV Gainor JF Heist RS. Lung cancer. Lancet. (2021) 398:535–54. doi: 10.1016/S0140-6736(21)00312-3 34273294

[3] Heon S Johnson BE. Adjuvant chemotherapy for surgically resected non-small cell lung cancer. J Thorac Cardiovasc Surg. (2012) 144:S39–42. doi: 10.1016/j.jtcvs.2012.03.039 22502967

[4] Burdett S Pignon JP Tierney J Tribodet H Stewart L Le Pechoux C. Adjuvant chemotherapy for resected early-stage non-small cell lung cancer. Cochrane Database Syst Rev. (2015) CD011430. doi: 10.1002/14651858.CD011430 25730344 PMC10542092

[5] Bradbury P Sivajohanathan D Chan A Kulkarni S Ung Y Ellis PM. Postoperative adjuvant systemic therapy in completely resected non-small-cell lung cancer: A systematic review. Clin Lung Cancer. (2017) 18:259–273 e8. doi: 10.1016/j.cllc.2016.07.002 28162945

[6] Custodio Carretero AB Garcia Saenz JA Gonzalez Larriba JL Bobokova J Calles Blanco A Hernando Trancho F. Adjuvant chemotherapy for early-stage non-small-cell lung cancer. Single-center experience and literature review. Clin Transl Oncol. (2008) 10:560–71. doi: 10.1007/s12094-008-0251-x 18796373

[7] Kris MG Johnson BE Berry LD Kwiatkowski DJ Iafrate AJ Wistuba II. Using multiplexed assays of oncogenic drivers in lung cancers to select targeted drugs. JAMA. (2014) 311:1998–2006. doi: 10.1001/jama.2014.3741 24846037 PMC4163053

[8] Mitsudomi T Morita S Yatabe Y Negoro S Okamoto I Tsurutani J. Gefitinib versus cisplatin plus docetaxel in patients with non-small-cell lung cancer harboring mutations of the epidermal growth factor receptor (WJTOG3405): an open label, randomized phase 3 trial. Lancet Oncol. (2010) 11:121–8. doi: 10.1016/S1470-2045(09)70364-X 20022809

[9] Zhong WZ Wang Q Mao WM Xu ST Wu L Shen Y. Gefitinib versus vinorelbine plus cisplatin as adjuvant treatment for stage II-IIIA (N1-N2) EGFR-mutant NSCLC (ADJUVANT/CTONG1104): a randomized, open-label, phase 3 study. Lancet Oncol. (2018) 19:139–48. doi: 10.1016/S1470-2045(17)30729-5 29174310

[10] Yue D Xu S Wang Q Li X Shen Y Zhao H. Erlotinib versus vinorelbine plus cisplatin as adjuvant therapy in Chinese patients with stage IIIA EGFR mutation-positive non-small-cell lung cancer (EVAN): a randomized, open-label, phase 2 trial. Lancet Respir Med. (2018) 6:863–73. doi: 10.1016/S2213-2600(18)30277-7 30150014

[11] Wu YL Tsuboi M He J John T Grohe C Majem M. Osimertinib in resected EGFR-mutated non-small-cell lung cancer. N Engl J Med. (2020) 383:1711–23. doi: 10.1056/NEJMoa2027071 32955177

[12] Leonetti A Minari R Boni L Gnetti L Verzè M Ventura L. Phase II, open-label, single-arm, multicenter study to assess the activity and safety of alectinib as neoadjuvant treatment in surgically resectable stage III ALK-positive NSCLC: ALNEO trial. Clin Lung Cancer. (2021) 22:473–7. doi: 10.1016/j.cllc.2021.02.014 33762169

[13] Kwak EL Bang YJ Camidge DR Shaw AT Solomon B Maki RG. Anaplastic lymphoma kinase inhibition in non-small-cell lung cancer. N Engl J Med. (2010) 363:1693–703. doi: 10.1056/NEJMoa1006448 PMC3014291 20979469

[14] Soda M Choi YL Enomoto M Takada S Yamashita Y Ishikawa S. Identification of the transforming EML4-ALK fusion gene in non-small-cell lung cancer. Nature. (2007) 448:561–6. doi: 10.1038/nature05945 17625570

[15] Solomon BJ Mok T Kim DW Wu YL Nakagawa K Mekhail T. First-line crizotinib versus chemotherapy in ALK-positive lung cancer. N Engl J Med. (2014) 371:2167–77. doi: 10.1056/NEJMoa1408440 25470694

[16] Camidge DR Kim HR Ahn MJ Yang JC Han JY Lee JS. Brigatinib versus crizotinib in ALK-positive non-small-cell lung cancer. N Engl J Med. (2018) 379:2027–39. doi: 10.1056/NEJMoa1810171 30280657

[17] Horn L Wang Z Wu G Poddubskaya E Mok T Reck M. Ensartinib vs crizotinib for patients with anaplastic lymphoma kinase-positive non-small cell lung cancer: A randomized clinical trial. JAMA Oncol. (2021) 7:1617–25. doi: 10.1001/jamaoncol.2021.3523 PMC8414368 34473194

[18] Peters S Camidge DR Shaw AT Gadgeel S Ahn JS Kim DW. Alectinib versus crizotinib in untreated ALK-positive non-small-cell lung cancer. N Engl J Med. (2017) 377:829–38. doi: 10.1056/NEJMoa1704795 28586279

[19] Zhou C Kim SW Reungwetwattana T Zhou J Zhang Y He J. Alectinib versus crizotinib in untreated Asian patients with anaplastic lymphoma kinase-positive non-small-cell lung cancer (ALESIA): a randomized phase 3 study. Lancet Respir Med. (2019) 7:437–46. doi: 10.1016/S2213-2600(19)30053-0 30981696

[20] Wang S Yu H Gan Y Wu Z Li E Li X. Mining whole-lung information by artificial intelligence for predicting EGFR genotype and targeted therapy response in lung cancer: a multicohort study. Lancet Digit Health. (2022) 4:e309–19. doi: 10.1016/S2589-7500(22)00024-3 35341713

[21] Goldstraw P Chansky K Crowley J Rami-Porta R Asamura H Eberhardt WE. The IASLC lung cancer staging project: proposals for revision of the TNM stage groupings in the forthcoming (Eighth) edition of the TNM classification for lung cancer. J Thorac Oncol. (2016) 11:39–51. doi: 10.1016/j.jtho.2015.09.009 26762738

[22] Sun JM Lira M Pandya K Choi YL Ahn JS Mao M. Clinical characteristics associated with ALK rearrangements in never-smokers with pulmonary adenocarcinoma. Lung Cancer. (2014) 83:259–64. doi: 10.1016/j.lungcan.2013.11.009 24300132

[23] Sahu A Prabhash K Noronha V Joshi A Desai S. Crizotinib: A comprehensive review. South Asian J Cancer. (2013) 2:91–7. doi: 10.4103/2278-330X.110506 PMC3876666 24455567

[24] Du X Shao Y Qin HF Tai YH Gao HJ. ALK-rearrangement in non-small-cell lung cancer (NSCLC). Thorac Cancer. (2018) 9:423–30. doi: 10.1111/tca.2018.9.issue-4 PMC5879058 29488330

[25] Rodig SJ Mino-Kenudson M Dacic S Yeap BY Shaw A Barletta JA. Unique clinicopathologic features characterize ALK-rearranged lung adenocarcinoma in the western population. Clin Cancer Res. (2009) 15:5216–23. doi: 10.1158/1078-0432.CCR-09-0802 PMC2865649 19671850

[26] Shaw AT Yeap BY Mino-Kenudson M Digumarthy SR Costa DB Heist RS. Clinical features and outcome of patients with non-small-cell lung cancer who harbor EML4-ALK. J Clin Oncol. (2009) 27:4247–53. doi: 10.1200/JCO.2009.22.6993 PMC2744268 19667264

[27] Shin SH Lee H Jeong BH Choi YS Shin MH Kim S. Anaplastic lymphoma kinase rearrangement in surgically resected stage IA lung adenocarcinoma. J Thorac Dis. (2018) 10:3460–7. doi: 10.21037/jtd.2018.05.131 PMC6051845 30069341

[28] Zhou JX Yang H Deng Q Gu X He P Lin Y. Oncogenic driver mutations in patients with non-small-cell lung cancer at various clinical stages. Ann Oncol. (2013) 24:1319–25. doi: 10.1093/annonc/mds626 23277484

[29] Choi H Paeng JC Kim DW Lee JK Park CM Kang KW. Metabolic and metastatic characteristics of ALK-rearranged lung adenocarcinoma on FDG PET/CT. Lung Cancer. (2013) 79:242–7. doi: 10.1016/j.lungcan.2012.11.021 23261227

[30] Wan Y Qian Y Wang Y Fang F Wu G. Prognostic value of Beclin 1, EGFR and ALK in non-squamous non-small cell lung cancer. Discovery Oncol. (2022) 13:127. doi: 10.1007/s12672-022-00586-y PMC9675885 36401689

[31] Liu SY Bao H Wang Q Mao WM Chen Y Tong X. Genomic signatures define three subtypes of EGFR-mutant stage II-III non-small-cell lung cancer with distinct adjuvant therapy outcomes. Nat Commun. (2021) 12:6450. doi: 10.1038/s41467-021-26806-7 PMC8575965 34750392

[32] Kris MG Gaspar LE Chaft JE Kennedy EB Azzoli CG Ellis PM. Adjuvant systemic therapy and adjuvant radiation therapy for stage I to IIIA completely resected non-small-cell lung cancers: american society of clinical oncology/cancer care ontario clinical practice guideline update. J Clin Oncol. (2017) 35:2960–74. doi: 10.1200/JCO.2017.72.4401 28437162

[33] Pignon JP Tribodet H Scagliotti GV Douillard JY Shepherd FA Stephens RJ. Lung adjuvant cisplatin evaluation: a pooled analysis by the LACE Collaborative Group. J Clin Oncol. (2008) 26:3552–9. doi: 10.1200/JCO.2007.13.9030 18506026

[34] Wang Y Wan Y Qian Y. Prognostic factors of IIIAN2 non-small-cell lung cancer after complete resection: A systemic review and meta-analysis. Comput Math Methods Med. (2021) 2021:1068090. doi: 10.1155/2021/1068090 34938347 PMC8687771

[35] Cheng YF Hung WH Chen HC Cheng CY Lin CH Lin SH. Comparison of treatment strategies for patients with clinical stage T1-3/N2 lung cancer. J Natl Compr Canc Netw. (2020) 18:143–50. doi: 10.6004/jnccn.2019.7353 32023528

[36] Gao F Li N Xu Y Yang G. Evaluation of postoperative radiotherapy effect on survival of resected stage III-N2 non-small cell lung cancer patients. Front Oncol. (2020) 10:1135. doi: 10.3389/fonc.2020.01135 32850322 PMC7399051

[37] van Meerbeeck JP Surmont VF. Stage IIIA-N2 NSCLC: a review of its treatment approaches and future developments. Lung Cancer. (2009) 65:257–67. doi: 10.1016/j.lungcan.2009.02.007 19285751

[38] Zhou K Zhao Y Liang L Cao J Lin H Peng Z. Adjuvant chemotherapy may improve long-term outcomes in stage IB non-small cell lung cancer patients with previous Malignancies: A propensity score-matched analysis. Front Oncol. (2022) 12:938195. doi: 10.3389/fonc.2022.938195 36119504 PMC9472252

[39] Boffa DJ Hancock JG Yao X Goldberg S Rosen JE Kim AW. Now or later: evaluating the importance of chemotherapy timing in resectable stage III (N2) lung cancer in the National Cancer Database. Ann Thorac Surg. (2015) Jan; 99(1):200-8. doi: 10.1016/j.athoracsur.2014.08.040 25440272

[40] Jung H-A Ku BM Kim YJ Park S Sun J-M Lee S-H. Longitudinal monitoring of circulating tumor DNA from plasma in patients with curative resected stages I to IIIA EGFR-mutant non–small cell lung cancer. J Thorac Oncol. (2023) 18:1199–208. doi: 10.1016/j.jtho.2023.05.027 37308037

[41] Behera M Steuer CE Liu Y Fernandez F Fu C Higgins KA. Trimodality therapy in the treatment of stage III N2-positive non-small cell lung cancer: A national cancer database analysis. Oncologist. (2020) 25:e964–75. doi: 10.1634/theoncologist.2019-0661 PMC7288644 31943520

[42] Martins RG D'Amico TA Loo BW Jr Pinder-Schenck M Borghaei H Chaft JE. The management of patients with stage IIIA non-small cell lung cancer with N2 mediastinal node involvement. J Natl Compr Canc Netw. (2012) 10:599–613. doi: 10.6004/jnccn.2012.0062 22570291

[43] Liu JW Li J Lin G Shang XQ. Analysis of recurrence patterns after curative resection of stage IIIA-N2 non-small cell lung cancer. Zhonghua Yi Xue Za Zhi. (2012) 92:2314–8. 23158558

[44] Liu J-W Li J Lin G Shang X-Q. Prognostic analysis of curative surgery for stage IIIA-N2 non-small cell lung cancer. Zhonghua zhong liu za zhi [Chinese J oncology]. (2013) 35:50–3. doi: 10.3760/cma.j.issn.0253-3766.2013.01.011 23648301

[45] Qiang G Guo Y Xiao F Yu Q Liang C Song Z. Analyses of risk factors for postoperative recurrence after curative resection of stage III A-N2 non-small cell lung cancer. Zhonghua Yi Xue Za Zhi. (2014) 94:3239–43. 25604225

[46] Peters S Bexelius C Munk V Leighl N. The impact of brain metastasis on quality of life, resource utilization and survival in patients with non-small-cell lung cancer. Cancer Treat Rev. (2016) 45:139–62. doi: 10.1016/j.ctrv.2016.03.009 27019457

[47] Zhang I Zaorsky NG Palmer JD Mehra R Lu B. Targeting brain metastases in ALK-rearranged non-small-cell lung cancer. Lancet Oncol. (2015) 16:e510–21. doi: 10.1016/S1470-2045(15)00013-3 26433824

[48] Ou SH Ahn JS De Petris L Govindan R Yang JC Hughes B. Alectinib in crizotinib-refractory ALK-rearranged non-small-cell lung cancer: A phase II global study. J Clin Oncol. (2016) 34:661–8. doi: 10.1200/JCO.2015.63.9443 26598747

[49] Gadgeel SM Gandhi L Riely GJ Chiappori AA West HL Azada MC. Safety and activity of alectinib against systemic disease and brain metastases in patients with crizotinib-resistant ALK-rearranged non-small-cell lung cancer (AF-002JG): results from the dose-finding portion of a phase 1/2 study. Lancet Oncol. (2014) 15:1119–28. doi: 10.1016/S1470-2045(14)70362-6 25153538

[50] Kodama T Hasegawa M Takanashi K Sakurai Y Kondoh O Sakamoto H. Antitumor activity of the selective ALK inhibitor alectinib in models of intracranial metastases. Cancer Chemother Pharmacol. (2014) 74:1023–8. doi: 10.1007/s00280-014-2578-6 25205428

[51] Tomasini P Egea J Souquet-Bressand M Greillier L Barlesi F. Alectinib in the treatment of ALK-positive metastatic non-small cell lung cancer: clinical trial evidence and experience with a focus on brain metastases. Ther Adv Respir Dis. (2019) 13:1753466619831906. doi: 10.1177/1753466619831906 30786826 PMC6385324

